# Study on the evaluation of rheumatoid arthritis via doppler ultrasonography and anti-cyclic citrullinated peptide antibody analysis

**DOI:** 10.12669/pjms.36.3.1492

**Published:** 2020

**Authors:** Li-na Shang, Gui-xin Di, Feng-ju Wei, Zheng Zhang

**Affiliations:** 1 Li-na Shang, Department of Ultrasound Imaging, Affiliated Hospital of Hebei University, Baoding, Hebei 071000, P.R. China; 2 Gui-xin Di, Department of Ultrasound Imaging, Affiliated Hospital of Hebei University, Baoding, Hebei 071000, P.R. China; 3 Feng-ju Wei, Department of Ultrasound Imaging, Affiliated Hospital of Hebei University, Baoding, Hebei 071000, P.R. China; 4 Zheng Zhang, Department of Rheumatology and Immunology, Affiliated Hospital of Hebei University, Baoding, Hebei 071000, P.R. China

**Keywords:** Anti-cyclic citrullinated, Peptide antibody, Rheumatoid arthritis, Ultrasonography

## Abstract

**Objective:**

This study aims to discuss the value of ultrasonic analysis and anti-cyclic citrullinated peptide (CCP) antibody analysis in evaluating the state of rheumatoid arthritis (RA).

**Methods:**

This study was conducted during March 2016 to December 2016. Total 82 patients with RA who sought treatment in the Affiliated Hospital of Hebei University were included in this study. Data on ultrasonic and anti-CCP antibody, ESR, and RF were collected and compared. The RA patients were divided into two groups of mild disease activity (DAS28 ≤ 3.2) and moderate-severe disease activity (DAS28 > 3.2) to compare the changes in synovial thickness of joints. The changes of joint ultrasonography were also compared between positive and negative anti-CCP antibodies group.

**Results:**

It is found that the number of patients suffering from joint involvement in the negative anti-CCP antibody group was larger than that of the anti-CCP positive antibody group (*P* <0.05); the thickness of the synovium of joints of patients in the group with moderate-severe disease activity evaluated via ultrasonography was significantly larger than that of the group with mild disease activity (*P* <0.05).

**Conclusion:**

It is possible to observe the degree of disease activity dynamically by combining ultrasonography with anti-CCP antibody and make a better assessment of patients to facilitate treatment.

## INTRODUCTION

Rheumatoid arthritis (RA) is a group of autoimmune diseases mainly featuring symmetrical synovitis changes of the joints. Arthritis and bone destruction may occur early in the course, and disabilities at different levels may be caused by disease progression if diagnosis and treatment are not given during the early stages. The standard examinations for the clinical diagnosis and disease evaluation are tests for anti-CCP antibodies in serum, rheumatoid factors (RF), and erythrocyte sedimentation rate (ESR).^1^ Joint examination methods include x-ray film, magnetic resonance imaging, and computed tomography. MRI and X-ray are more commonly used in imaging diagnosis. In recent years, color Doppler ultrasonography is also widely used in the examination and evaluation of RA, but ultrasound needs more careful analysis in the diagnosis and disease assessment of RA.^2^ The sensitivity of anti-CCP antibodies to RA is not high, but the specificity is strong. At the same time, anti-CCP antibodies can assess the progress of RA to a certain extent.^3^ It is an important direction to combine ultrasound assessment and anti-CCP antibody assessment from different angles to diagnose and evaluate RA. This study aims to discuss the effect of applying color Doppler ultrasonography and anti-CCP antibodies in evaluating the severity of RA patients.

## METHODS

### Clinical data

Eighty-two patients with RA sought treatment at the Affiliated Hospital of Hebei University from March 2016 to December 2016. The individuals who were chosen consisted of 82 male patients aged 30 to 69 years. All patients complied with the 2010 RA classification criteria of the CRA, the Chinese Rheumatology Association.^1^

### Ethical approval

The study was approved by the Institutional Ethics Committee of the Affiliated Hospital of Hebei University, and written informed consent was obtained from all participants.

The patients received a laboratory serologic examination for anti-CCP antibody, ESR, and RF. The anti-CCP antibody test method that involved collecting 2 ml of venous blood on an empty stomach, separating the serum after centrifugation, and then performing an enzyme-linked immunosorbent assay (ELISA;<20 RU/ml means negative, and >20 RU/ml means positive). CRP and RF also were tested with ELISA. Disease activity (DAS28 score) was calculated according to clinical symptoms, physical signs, and test results. DAS28 = [0.56 × (tender joint count) ½ ± 0.28 × (swollen joint count) ½ ± 0.70 In(ESR)] X 1.08 ± 0.16. The RA patients were divided into a group with positive anti-CCP antibodies and a group with negative anti-CCP antibodies based on the test results of anti-CCP antibodies to compare the changes of joint ultrasonography. According to the disease activity determined based on the DAS28 scoring standards, the RA patients were divided into a group with mild disease activity (DAS28 ≤ 3.2) and one with moderate-severe disease activity (DAS28 > 3.2) to compare the changes in synovial thickness of wrist joints, metacarpophalangeal joints, proximal interphalangeal joints, knee-joints, and ankle joints tested via ultrasonography between the two groups.

### Ultrasonography

All patients received ultrasonography of their wrist joints, metacarpophalangeal joints, proximal interphalangeal joints, elbow joints, knee-joints, and ankle joints. A VVE9 high-frequency probe with a frequency of 10~15 MHz manufactured by GE was employed, and the instrument was operated by experienced sonographers. The affected joint count and presence of arthroedema, synovial pannus formation, or bone erosion were recorded, and synovial thickness was tested. If one or more of the indexes mentioned above was detected, it indicated joint involvement, with at least a small amount or more of arthroedema. If the synovial membrane of the facet joint bulged above the highest ligature of the two bones and the synovial thickness of the large joint was more substantial than 0.2 mm, it should be deemed as synovial thickening. symptoms of bone erosion included cracks or coloboma on the bone surface and uneven surface; synovial pannus meant that the synovial membrane covering the cartilage surface of the joint was displayed as a color flow signal on Doppler. For purposes of this study, a punctiform, dotted-line, dendritic, or reticulate flow signal meant positive.

### Statistical analysis

SPSS 22.0 statistical software was used for statistical analyses. The measurement data was expressed in the form of the mean value ± standard deviation. A *t*-test was conducted for comparison among groups, the chi-square test was used for comparison among groups of categorical data, and *P* <0.05 meant that the difference was of statistical significance.

## RESULTS

The anti-CCP antibodies and RF of the group with positive anti-CCP antibodies were significantly higher than that of the group with negative anti-CCP antibodies, and the difference was of statistically significant (*P* <0.05). The comparison of the synovial thickness of joints, ESR, and DAS28 score between the two groups showed that the difference was not statistically significant (*P* >0.05). Based on the test results of anti-CCP antibodies, the number of patients in the group with positive anti-CCP antibodies was 42, and that in the group with negative anti-CCP antibodies was 40. Ultrasonic examination results showed that the numbers of affected joints of the two groups were: wrist joint: 38 vs. 35, metacarpophalangeal joints: 64 vs. 120, proximal interphalangeal joint: 43 vs. 45, elbow joint: 1 vs. 1, knee joint: 11 vs. 0, and ankle joint: 3 vs. 0. The total numbers of affected joints of the two groups were 160 and 201, respectively. Ultrasonic examination results of the group with positive anti-CCP antibodies showed that among the patients with joints affected, 8 patients suffered from arthroedema, one patient from bone erosion, and 27 patients from synovial pannus formation. Ultrasonic examination results of the group with negative anti-CCP antibodies showed that among the patients with joints affected, two patients suffered from arthroedema, no one from bone erosion, and no patients from synovial pannus formation. (Table-I)

**Table-I:** Laboratory examination results patients with RA with positive anti-CCP antibodies and with negative anti-CCP antibodies.

Index	Group with positive anti-CCP antibodies (42)	Group with negative anti-CCP antibodies (40)	P-value
Anti-CCP antibodies	125±75	9±5	0.025*
RF(IU/L)	189±72	89±38	0.031*
Synovial thickness of joint (mm)	2.5±1.3	2.3±0.4	0.106
ESR(mm/h)	48±18	50±23	0.251
DAS28 score	3.5±0.6	3.5±0.3	0.451
Wrist joint	38	35	
Metacarpophalangeal joint	64	120	
Proximal interphalangeal joint	43	45	
Elbow joint	1	1	
Knee-joint	11	0	
Ankle joint	3	0	
Total number of affected joints	160	201	
Arthroedema	8	2	
Bone erosion	1	0	
Pannus formation	27	15	

* p<0.05 CCP: cyclic citrullinated peptide;

DAS28: Disease Activity Score for Rheumatoid Arthritis.

Among the 82 RA patients were grouped by disease activity, 28 patients had DAS28 ≤ 3.2, indicating mild disease activity; 54 patients had DAS > 2.8, indicating moderate-severe activity. The comparison of anti-CCP antibodies between the two groups had no statistical significance [(76 ± 46) RU/ml, (96 ± 36) RU/ml, *P* >0.05]. Ultrasonic examination results showed that the synovial thickness of the joints of the group with moderate-severe disease activity was significantly larger than that of the group with mild disease activity, and the difference was statistically significant [(2.7 ± 1.2) mm, (2.1 ± 0.7) mm, *P* <0.05]. (Table-II).

**Table-II:** Laboratory examination results of patients of with mild disease activity and of with moderate-severe disease activity.

Index	Mild disease activity (DAS28≤3.2)	Moderate-severe disease activity DAS28>3.2	P-value
Patients	28	54	
Anti-CCP antibodies (RU/ml)	76±46	96±36	0.061
Synovial thickness of joint (mm)	2.1±0.7	2.7±1.2	0.025*

* p<0.05, CCP: cyclic citrullinated peptide;

DAS28: Disease Activity Score for Rheumatoid Arthritis.

## DISCUSSION

High-frequency ultrasonography has several advantages, such as no radiation, noninvasive, repeatability, convenience, and economic efficiency; it is also efficient in reflecting patients’ anatomical structure and surrounding tissues. In the new RA classification criteria issued by ACR/EULAR in 2010, ultrasonography was listed as a method of determining synovitis for the first time.^4^ In recent years, its application in rheumatic diseases, especially in joint diseases, has attracted much attention. Anti-CCP antibodies are widespread in synovial tissues of the joints of RA patients. They are the main target antigens of the autoimmune response, of which the titer is related to disease activity.^5^ The comparison between the groups divided by disease activity showed no statistically significant difference (*P* > 0.05) In contrast, ultrasonic examination results showed that the synovial thickness of joints of patients of severe activity was significantly larger than that of the group with mild activity with a statistically significant difference (*P* < 0.05), indicating that the synovial thickness of joints is independent of anti-CCP antibodies, but is positively related to disease activity. This finding is of great guiding significance for RA treatment. Clinically, patients’ medication should be based on clinical symptoms, ESR, CRP, and Doppler ultrasonic examination results (especially power Doppler ultrasonography) to ensure scientific and rigorous treatment. Recent studies have also shown that certain types of ultrasound and MRI have almost the same accuracy in the diagnosis and evaluation of RA. It is necessary to use ultrasound and anti CCP antibody to evaluate RA in the future.^6^

By affected joint count detected via ultrasonography in this study, the affected joint count (201) (including wrist joint, metacarpophalangeal joints, and proximal interphalangeal joint) of the group with negative anti-CCP antibodies is significantly larger than that of the group with positive anti-CCP antibodies (160). Although anti-CCP antibodies are of high specificity and sensitivity and are vital to diagnosing RA, some patients will turn out to be negative in anti-CCP antibodies. In such a case, color Doppler ultrasonography can be used to observe the condition of the synovium of the joint, affected joint count, and bone erosion of patients, depending on which of the affected joint counts and locations can be displayed accurately.^7^

RA is a common disease among middle-aged groups, and especially among women. It is a chronic inflammatory synovial disease involving multiple joints and organs. The early pathological change of RA is chronic synovitis, namely synovial membrane oozes and incrassates. Generally, the synovial membrane thickness is not larger than 2 mm. The synovial membrane thickness of RA patients is above 2 mm. The synovial membrane thickness of all RA patients in the study is also above 2 mm. Synovial membrane incrassating is a sensitivity index for diagnosing this disease.^8^,^9^ Ultrasonography works well in distinguishing soft tissue and is a sensitive method of displaying changes in the synovial membrane. There are also studies showing that ultrasonography frequency should be adjusted according to the amount of anti-CCP antibodies, and the two indexes become increasingly related to each other.^10^,^11^ Anti-CCP antibodies widely exists in the synovial tissues of joints of RA patients as the main target antigen of the autoimmune response, of which the titer is relating to disease activity.^12^,^13^ Studies have shown that anti-CCP antibodies are an independent predictive factor of the involvement of the myocardium of patients with active RA, indicating that anti-CCP antibodies should be further studied in the future to help make better diagnoses and implement subsequent treatment strategies.^14^ In the pathogenetic process of RA, blood vessel hyperplasia of the synovial membrane occurs early, and even exists before the occurrence of the characteristic and histological symptoms. Twenty-seven patients in the study suffered from pannus formation, which is thought to be involved with neovascularization, hyperplasia of the synovial membrane, a significant increase in hemoperfusion, and pannus formation. This study shows that ultrasonography and the anti-CCP antibody test are of great significance for evaluating RA and monitoring disease activity.^15^

## CONCLUSION

Previous studies have shown that the methods used by some investigators for diagnosing RA via ultrasonography have a complicated scoring process that is subject to different criteria and limited by the operator’s technical level. As the technology matures, however, standardized ultrasonography, RA evaluation system will be established, and ultrasonography will play a major role combined with the anti-CCP antibody in diagnosing and treating RA in the future.

### Authors’ Contributions:

**LS and GD** designed this study and prepared this manuscript.

**ZZ** collected and analyzed clinical data and is responsible and accountable for the accuracy or integrity of the work.

**FW** significantly revised this manuscript.
